# C-Arm Image-Based Surgical Path Planning Method for Distal Locking of Intramedullary Nails

**DOI:** 10.1155/2018/4530386

**Published:** 2018-05-23

**Authors:** Wei-En Hsu, Ching-Hsiao Yu, Chih-Ju Chang, Hung-Kang Wu, Tsong-Han Yu, Ching-Shiow Tseng

**Affiliations:** ^1^Department of Mechanical Engineering, National Central University, Taoyuan 32001, Taiwan; ^2^Department of Orthopedics, Taoyuan General Hospital, Taoyuan 33004, Taiwan; ^3^Department of Neurosurgery, Cathay General Hospital, Taipei, Taiwan; ^4^Department of Medicine, School of Medicine, Fu Jen Catholic University, New Taipei City, Taiwan

## Abstract

Due to the curvature of the bone marrow cavity, the intramedullary nail used in long bone fracture fixation can be deformed, causing displacement of the locking holes. In this study, an algorithm using only one C-arm image to determine the center positions and axial directions of locking holes was developed for drilling guidance. Based on conventional method that the axial direction of locking hole would be identified when locking hole contour is presented as a circle, the proposed method can locate the circle contour centroid by using one C-arm image including two elliptical contours. Then the two distal locking holes' axial direction and centers would be determined. Three experiments were conducted to verify the performance of the proposed algorithm, which are (1) computer simulation, (2) use of real intramedullary nails, and (3) actual drilling test with the bone model. The experimental results showed that the average error of the axial direction and center position were 0.62 ± 0.6°, 0.73 ± 0.53 mm (simulation) and 3.16 ± 1.36°, 1.10 ± 0.50 mm (actual nail), respectively. The last ten drilling test sets were completed successfully (with an average duration of 48 seconds). Based on the experimental results, the proposed algorithm was feasible for clinic applications.

## 1. Introduction

**Figure 1:**
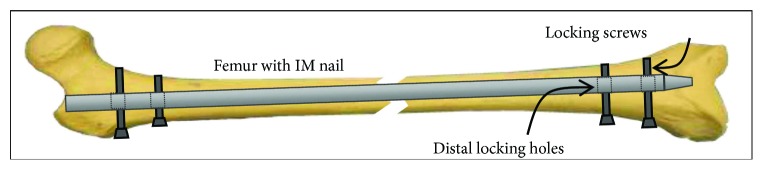
Illustration of an implanted intramedullary nail inside a femur.

In long bone fracture fixation, the use of intramedullary nails is one of the recommended treatments. Intramedullary nail is implanted into the bone marrow cavity and fixed with locking screws at both ends (Figure 1). Once a nail is implanted into the bone marrow cavity of a long bone, a surgical drill is used to drill the bone through the locking hole, and a locking screw is then locked to fix the nail and the bone. Due to the particular shape of the bone marrow cavity, bending of the intramedullary nail can occur during the process of implantation, causing displacement of the locking hole, which results in the auxiliary drilling guide not being properly aligned with the locking hole [1]. This issue remains a major problem in the distal locking of intramedullary nails.

**Figure 2:**
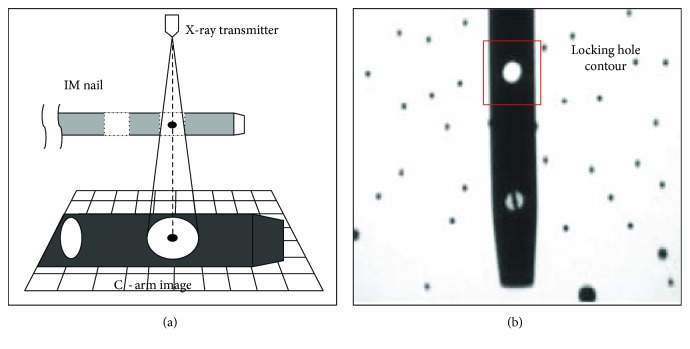
(a) Schematic of circular contour imaging of the locking hole. (b) Circular contour image of the locking hole.

The conventional distal locking method for intramedullary nails involves the use of C-arm X-ray imaging to identify the direction and location of the locking holes. The process requires the surgeon to adjust the projection direction of the C-arm X-ray constantly until a circular locking hole image is projected. If the locking hole image is presented as a circle, it indicates a near parallel direction between the axis of the locking hole and the central axis of the cone-shaped X-ray projection, allowing the surgeon to ascertain the axis of the locking hole (Figures 2(a) and 2(b)). Based on the surgeon's personal clinical experience, he or she will be able to drill the bone along the axial direction of the locking hole by trial and error approach. However, such a method incurs the following problems:

1. Adjustment of the C-arm X-ray projection direction and location continuously can be difficult and time consuming during the locking hole positioning, drilling and tightening of the locking screws. Moreover, a high radiation dosage due to continuous X-ray exposure that is harmful to medical staff and the patient.
2. Overdrilling can also occur, or the drill bit can be broken, resulting in locking failure and a poor surgical quality.

Therefore, the aim of this study was to propose an algorithm for fast and accurate calculation of the center location and axial direction of distal locking holes based on a single C-arm image. In previous years, many approaches have been proposed to solve the distal locking problem encountered with the use of a guidance jig, which can be generally summarized as the use of improved guidance mechanisms [2–8], electromagnetic fields [9], and image guidance methods [10–14]. As mentioned above, due to nail deformation, the guidance jig is considered unreliable and is rarely used clinically. The electromagnetic field method requires implantation of a field sensor into the intramedullary nail, but the narrow inner space makes it difficult to place the sensor at the correct location, let alone in alignment with the axial direction of the locking hole. Image guidance method is based on C-arm images of the nail to identify the locking hole centers and axial directions. Zhu et al. [15] identified the axial direction of the nail by its AP and lateral C-arm images. Through contour image analysis and iterative computation, the rotation angle of the nail was calculated. Both values were combined to define the location and direction of the locking hole. Neatpisarnvanit and Suthakorn [16] used the aspect ratio of the projected image contour of the locking hole as a basis to establish the baseline value for locking hole axis direction evaluation. However, the method assumed that the axial direction of the intramedullary nail and the normal of the C-arm image plane were parallel, upon which the correlation between self-rotation and the contour change of the locking hole was analyzed and established based on the fixed distance between the nail and the C-arm X-ray transmitter, making this method inapplicable in real surgical practice. Yaniv and Joskowicz [17], on the other hand, developed a distal fixation guidance system on a Stewart platform. An X-ray image of the intramedullary nail was used for 3D pose estimation algorithm of the locking hole axis. By defining feature points of the intramedullary nail, its possible position and orientation were calculated from the projected image, even though the method partially relied on precalibration of the C-arm image taken at an appropriate location and reimaging during the procedure, which elongated the duration of surgery. Esfandiari et al. [18] first set a positioning marker on the C-arm and used biplane C-arm X-ray images for spatial positioning, along with image analysis to specify the feature points of the intramedullary nail and complete its 3D pose estimation. Thus, the method was similar to those developed in previous research, in that two images were required, and feature points were identified in advance, resulting in a relatively longer operation duration. Based on C-arm image surgical navigation technique [19], this study proposed an innovative method by which a single X-ray image of the intramedullary nail is used to calculate the center location and axial direction of the locking hole, which the surgeon can then use as a guidance for drilling. After the direction and location of the locking hole had been calculated, the tip position and axial direction of the drill tracked by using an optic tracker (Spectra, Northern Digital Inc., Canada) could be adjusted to enable drilling along the axial direction of the locking hole.

## 2. Materials and Method

The drilling path-planning algorithm for distal locking of an intramedullary nail based on the center location and axial direction of the locking hole employed an intraoperative C-arm image. The main procedure included C-arm image calibration, image feature segmentation, establishment of the C-arm X-ray projection model, and calculation of the center position and axial direction of the locking hole. The C-arm image was obtained using a C-arm system (Solo, Ziehm Imaging Inc.) and captured by an image capture card (Epiphan Systems Inc.); the resolution of the captured image was 1280 × 720 pixels.

### 2.1. C-Arm Image Projection and Correction

**Figure 3:**
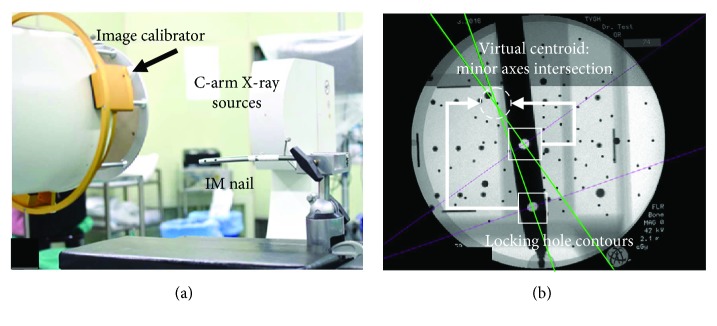
(a) Experimental setup. (b) C-arm image of the distal locking holes.

**Figure 4:**
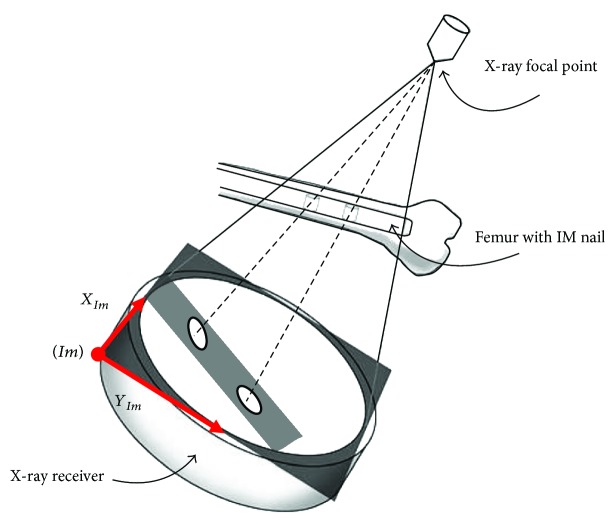
Definition of the image coordinate system.

According to the studies of Fahrig et al. [20] and Gorges et al. [21], the image projection mode of the C-arm system was similar to the single point perspective projection, and the path-planning algorithm was designed in this study based on that premise. However, as the image of intensifier-typed C-arm (Solo, Ziehm) had pincushion distortion and S-shaped sigmoidal distortion [22], it was necessary to perform image correction [23] to acquire a distortion-free C-arm image. This distortion correction step can be skipped in case a flat panel C-arm is used. The main concept of C-arm image correction was to compute and correct the image distortion with an image calibrator device mounted on X-ray receiver (Figure 3(a)). The image calibrator was a double layer device with steel balls as features, and the features' spatial positions and projected contour centroids were used in image distortion correction and X-ray focal point calculation. The distortion model [*F*_dist_] = [*M*][*F*_cort_] was formed, where [*F*_dist_] and [*F*_cort_] matrixes contain steel balls' distorted image contour centroids and their ideal centroids based on image calibrator geometry and the mapping matrix [*M*] formed by cubic polynomial equation (20 coefficients). Using the spatial coordinates of the corrected image contour centroids and physical positions of the feature points on the second layer, the C-arm projection geometry can be calculated. In our case, the image plane position with respect to the image calibrator is known by design. This has two consequences. First, any pixel location can be readily translated into a 3D point position. And second, the only unknown in the C-arm geometry is the position of the focal point *f* which can be obtained by the inverse projection of features on second layer. An illustration of the relative positions in the image coordinate system (*Im*) of the X-ray focal point *f* and the intramedullary nail is shown in Figure 4.

### 2.2. Imaging Characteristics of the Distal Locking Hole Contour

**Figure 5:**
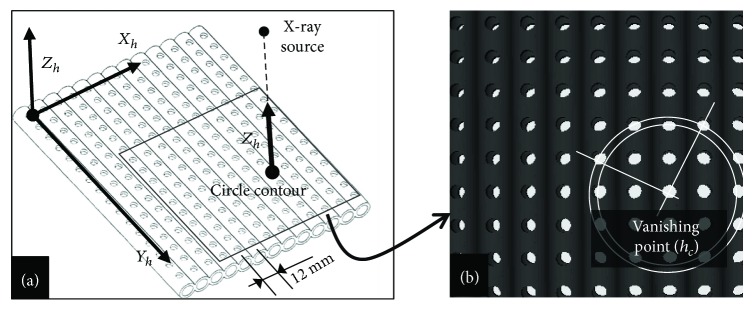
(a) Simulation model. (b) Imaging pattern.

The locking hole has the shape of a right cylinder. Its image contour is then formed by the intersection of the projection of both its circular bases (see Figure 5). It can be thought of as an approximate ellipse whose small axis is the projection of the cylinder axis. If the image contour of the locking hole is a circle, this means that the axis of the locking hole is identical to the central axis of the X-ray projection model. To illustrate and summarize the relationship between the projected image and the axial direction of the locking hole, an image projection simulation program of a locking hole was designed based on the C-arm projection model. The nail computer model was of the actual size of an intramedullary nail (Stryker Corporation), with an external diameter of 13 mm and a hole diameter of 5 mm. As shown in Figure 5(a), the locking holes were arranged at an interval of 12 mm and were in alignment with the axial direction *Y*_*h*_ of the nail. Thirteen nails were arranged side by side on a plane (the *X*_*h*_ direction), and thus the *Z*_*h*_ direction was also the axial direction of any of the locking holes. Figure 5(b) presents the simulated C-arm projection image of parallel and coplanar nail models of Figure 5(a). It can be seen from the two figures that since all locking hole axes are parallel, these axes project on the X-ray image onto a set of lines that intersect at a common vanishing point *h*_*c*_. According to the assumptions above, this point can therefore be determined in practice as the intersection of the small axes of both locking holes projection images. The direction *Z*_*h*_, common to both locking holes, is thereafter determined as leading the line passing through *f* and *h*_*c*_.

### 2.3. Planning of the Drilling Pathway of the Locking Hole

#### 2.3.1. Calculation of the Axial Direction of the Locking Hole

**Figure 6:**
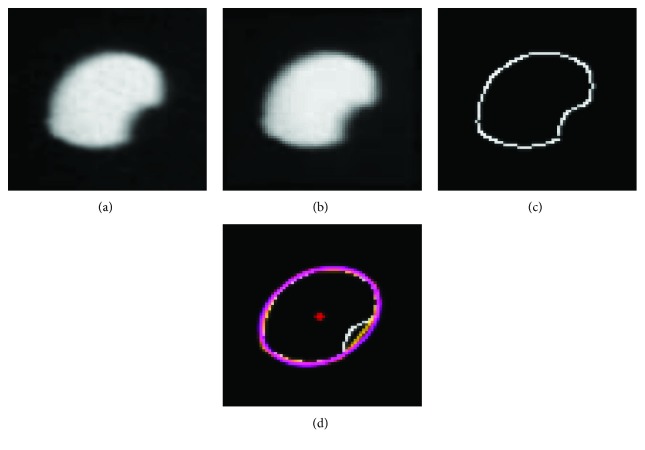
Segmentation of the locking hole contours: (a) raw image; (b) enhanced image; (c) detected raw contour; and (d) fixed contour.

**Figure 7:**
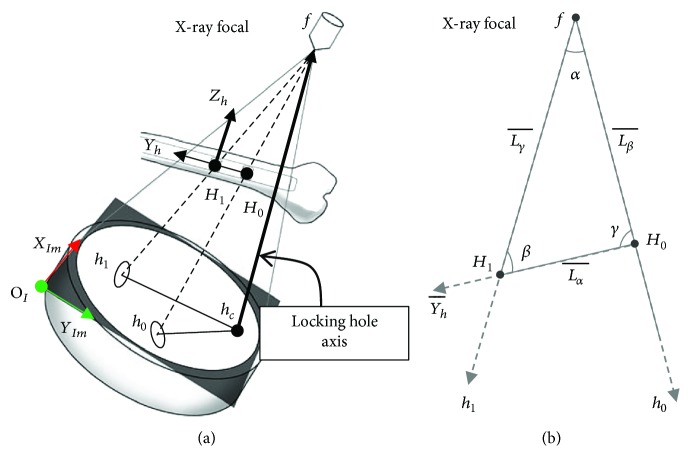
(a) Relative spatial relationship. (b) Geometric diagram on the *M* plane.

Based on the imaging characteristics of the locking hole described above, the algorithm for determining the axial direction of the locking hole can be described as follows:

1. A C-arm image of the two locking holes was taken. Of the two image contours of the two locking holes, it would be a circle and an approximate ellipse or both approximate elliptical contours.
2. After acquisition of the C-arm X-ray image (Figure 6(a)), the two image contours of the locking holes were applied by closing operation (dilation followed by erosion) for noise reduction and edge enhancement (Figure 6(b)). Using the Canny method to obtain the raw contours (Figure 6(c)), the defects of the two original contours due to image noise or blocking of steel markers of the image calibrator were fixed using the convex hull algorithm [24] (Figure 6(d)).
3. The repaired contours were matched to the mathematical description by the elliptical matching technique [25], and its centroids as well as the major and minor axes of the detected contours were calculated.
4. If either of the two elliptical contours (*h*_0_, *h*_1_) was determined to be an approximate circle, *h*_*c*_ was defined at the circle centroid.
5. If the two image contours were not circular, then, for each of them, the straight line passing through the image contour centroid and parallel to the minor axis of the contour was established. *h*_*c*_ was defined as the intersection of the two minor axes.
6. Finally, the axial direction of locking holes (*Z*_*h*_ in Figures 5(a) and 7) could be determined by connecting *h*_*c*_ (determined in step (4) or (5)) to the X-ray focal point *f* (showed in Figure 5(a)).

According to the algorithm simulation experiment, the pixel number within the image contours of the two locking holes in step (2) must be larger than the preset threshold of 150 pixels. Also, the intersection angle between the minor axes of the image contours of the two locking holes must be larger than 10 degrees. These two conditions were established to prevent calculation error in the determination of the minor axes of the image contours and the axial directions of the locking holes. In step (4), a self-developed method was used which would find a minimal fitting circle of hole image contour and calculate the fitness of each edge pixel to evaluate the circle similarity value. If the computed similarity value was larger than the criterion value (0.7) based on simulation experimental result, the image contour is considered as a circle.

#### 2.3.2. Calculation of the Locations of the Locking Hole Centers

Planning of the drilling pathway for distal locking of the intramedullary nail also required identification of the locking hole centers. As illustrated in Figure 7(a), the known geometric data of the C-arm X-ray projection model of the intramedullary nail included the location of the ideal X-ray focal point *f* relative to the image plane, the line segment $\bar{L_{\alpha }}$ between the centers of the two locking holes, and the centroids of the images contours of the locking holes. Then, the projection rays of locking holes were established, and by the Law of Cosines, angle *α* could be calculated. For a small cone angle of projection, the projection of the center of the locking hole could be thought as at the elliptical contour centroid of the locking hole image [26], and thus, projection ray that connected *f* and the image contour centroid of *h*_0_ would pass through the center of *H*_0_. Similarly, the projection ray of *h*_1_'s centroid would pass through the center of *H*_1_. Also, the line segment $\bar{L_{\alpha }}$, as the axis of the intramedullary nail, must be on plane *M*, which was determined from *f*, centroid of *h*_0_ and *h*_1_. Therefore, the normal vector $\overset{⃑}{N_{m}}$ of plane *M* could be calculated as the cross product of projection rays. Then, the axis *Y*_*h*_ of the intramedullary nail coordinate system was perpendicular to the normal vector $\overset{⃑}{N_{m}}$ and the *Z*_*h*_ axis of the intramedullary nail coordinate system (axis of the locking hole). Therefore, the direction of *Y*_*h*_ could be defined as cross product of $\overset{⃑}{N_{m}}$ and *Z*_*h*_.

To calculate distal locking hole centers of *H*_0_ and *H*_1_, the length and direction of known segment $\bar{L_{\alpha }}$ (calculated direction of *Y*_*h*_) and the plane *M* could be used. As shown in Figure 7(b), in the Euclidean geometry, the focal point and two locking hole centers formed a triangle. The side length of the triangle $\bar{L_{\alpha }}$ was known (the distance between the two locking holes), and its corresponding angle *α* was also known. The other two angles (*β* and *γ*) could be calculated with vectors $\overset{⃑}{Y_{h}}$ and projection rays of two hole contour centroids by the Law of Cosines. With known angles and a corresponding side, by the Law of Sines, the lengths of the remaining two sides ($\bar{L_{\beta }}$ and $\bar{L_{\gamma }}$) could be calculated. Finally, based on calculated distance and direction, the center positions of two locking holes could be calculated.

### 2.4. Experiments

The drilling pathway-planning algorithm for distal locking of the intramedullary nail was first tested in a software simulation. Second, the actual intramedullary nail C-arm image was used. Finally, the implant of an intramedullary nail inside the sawbone model was imaged by the C-arm equipment. By applying the algorithmic calculation described above, the location and direction of the locking holes were obtained before commencing drilling under guidance for outcome confirmation.

#### 2.4.1. Simulation for Positioning of the Virtual Intramedullary Nail Axis and the Axial Direction

The simulation program reconstructed a C-arm X-ray perspective projection environment in the virtual space according to a real C-arm projection mode (Solo, Ziehm Inc.). The location and direction of the 3D model of a physical intramedullary nail were specified in the virtual space. The program automatically generated a projected image of the locking hole for testing. The test cycle included the following steps: (1) generate the position and direction of nail model (correct solution); (2) simulate the C-arm projection image; (3) perform segmentation of the image contours of the intramedullary nail and the two locking holes; (4) place the C-arm image and the contour data into the pathway-planning algorithm for calculation of the axis direction and center position of the locking hole to calculate its position and axial direction; and (5) record the positioning and directional error and complete the test. After completion of testing, statistical analysis was performed, and the algorithm stability and reliability was evaluated. And at first step, according to the clinical practice, the distance between intramedullary nail and C-arm X-ray receiver plane varied from 20 mm to 500 mm with an interval of 10 mm. Then the transverse (x, y) positions and directions of the locking holes were randomly generated inside the C-arm cone beam to ensure hole contours visible. Almost 500 test cases were generated at each interval to cover the C-arm cone beam volume.

#### 2.4.2. Experiment to Investigate the Actual Position of the Intramedullary Nail Axis and the Axial Direction

In order to further prove the clinical practicality of the algorithmic theory, a simulated distal locking experiment of intramedullary nail was designed in this study, as shown in Figure 3(a). To verify the algorithm for calculation of the center position and the axis of the locking holes, the physical intramedullary nail was fixated on a bed, as is the case during surgery. First, the optical tracking device and the positioning probe were used in an auxiliary equipment to identify the center locations and axial directions of the two locking holes of the intramedullary nail. Then, an X-ray image of the intramedullary nail was obtained. After using the algorithmic calculation to obtain the minor axial directions of the corrected and segmented images of the two locking holes, the virtual centroid was also calculated (Figure 3(b)). Thus, the axial directions and center positions of the locking holes were computed. Lastly, the differences between the measured and calculated axial directions and center positions of the locking holes were compared. In each sawbone experiment (including drilling test described below), locking hole images were taken and acquired at different distances between intramedullary nail and X-ray receiver. Moreover, at each distance, most of acquired image contours were approximate ellipses and their positions were distributed at uniform as possible.

#### 2.4.3. Experiment with Actual Guided Drilling of the Intramedullary Nail

**Figure 8:**
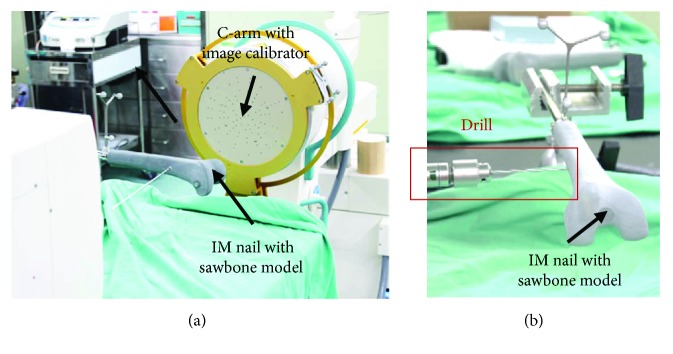
Experimental conditions of guided drilling.

The final verification phase of the algorithmic calculation involved the use of an intramedullary nail in a sawbone model. The surgeon, using a navigation software with the algorithm, would commence drilling. The experimental setting was similar to that described for the previous experimental phase, but the intramedullary nail was inserted in a sawbone model. The experimental environment was as shown in Figure 8. After calculation of the axial directions and center positions of the locking holes, the data were then sent to and displayed on the guidance system, on which the surgeon would base to commence drilling through the locking hole without seeing the actual intramedullary nail. The success or failure of drilling and the process duration were recorded in order to assess whether the method met the clinical demands.

## 3. Results and Discussion

**Figure 9:**
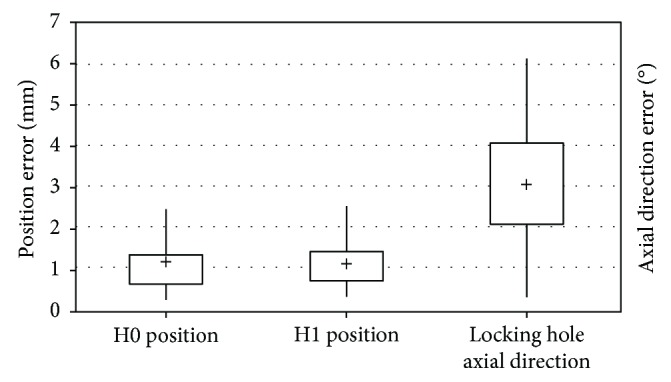
Position and direction error distributions of actual test.

A total of approximately 21,000 cases were tested in the algorithmic simulation, which were generated in such a way that the directions and positions of the intramedullary nails were produced randomly at different heights from the receiver end of the C-arm. The test results showed that the average distance error between the preset value and the calculated value of the center of the locking hole was 0.73 ± 0.53 mm. The average error of the axial direction of the locking hole was 0.62 ± 0.6°. The error components of the *X*_*h*_ and *Y*_*h*_ directions (as shown in Figure 7(a)) were 0.13 ± 0.05 mm and 0.13 ± 0.22 mm, respectively. For the *Z*_*h*_ direction, the error component was 0.48 ± 0.75 mm. It was found that the positioning error for the center was primarily in the *Z*_*h*_ direction, while there was a lesser impact on the positional guidance for drilling. As the positioning errors in the *X*_*h*_, *Y*_*h*_, and *Z* directions were rather small, it could be inferred that the method of positioning of the centers and axial directions of the locking holes for the intramedullary nail implantation could rely on a single C-arm image. Then, in testing of actual intramedullary nail imaging, X-ray images of the intramedullary nail at different heights from the receiver end of the C-arm were taken. A total of 51 cases were sampled (102 locking hole contours). The average spatial position error was 1.10 ± 0.50 mm, and the direction error was 3.16° ± 1.36°. The overall error distribution was as shown in Figure 9.

**Figure 10:**
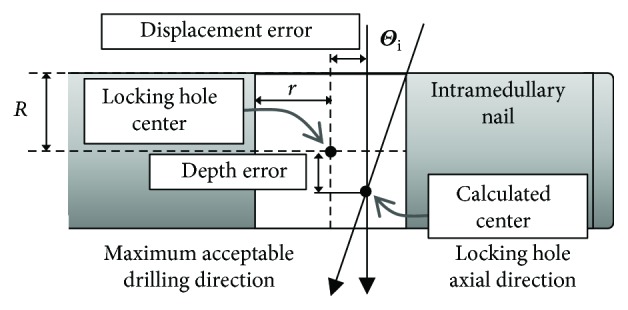
Maximum acceptable drilling direction and maximum acceptable angle.

Data of the error for drilling to the center can be classified as depth error (error along axial direction or *Z*_*h*_ direction of the locking hole) and displacement error (the distance deviation on the *X*_*h*_-*Y*_*h*_ plane of locking holes). By inputting both errors into the following equation, the maximum acceptable angle misalignment Θ_*i*_, corresponding to the depth and displacement errors, could be calculated:

(1) $$\begin{array}{c} \Theta_{i}=\tan^{-1}\left(\frac{r-displacement\;error}{R+depth\;error}\right), \end{array}$$

where *r* = the radius of the locking hole and *R* = the radius of the intramedullary nail (showed in Figure 10).

If the calculated angle misalignment was smaller than the maximum acceptable value, then drilling success could be expected. In the experiment, the errors of deviation in the spatial positioning and directional angle of all tested cases were smaller than the maximum acceptable angle for drilling, and the drill bit of 3 mm in diameter easily passed through the distal locking hole of the intramedullary nail of 5 mm in diameter.

**Figure 11:**
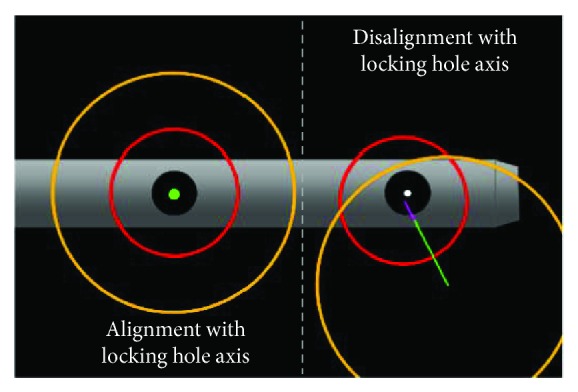
Drilling guidance display of the navigation system.

**Figure 12:**
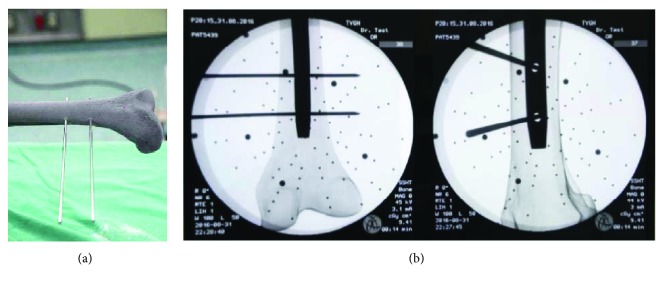
(a) Picture of the guided drilling outcome; (b) X-ray images of guided drilling (AP & Lateral).

In the final experiment, after C-arm imaging and calculation of center position and axial direction of locking holes, the existing surgical navigation software developed by our laboratory (real-time tracking of the drill tip and its axial direction) was used to guide a surgeon to commence drilling according to the guidance display, as shown in Figure 11. On the right side of the figure is presented a picture of the unaligned drill bit viewed from the axial direction of the locking hole, in which is shown the drill tip (center of the red circle), the drill tail (center of the orange circle), and the relative relationship between the drill tip axis (green line with purple tip highlight) and the locking hole. After the surgeon had aligned the drill axis with the locking hole axis, the display was shown as two concentric circles, as shown in the left figure in Figure 11. The surgeon would only have to maintain the course for successful drilling. The experiment was conducted in a total of 10 cases of 20 guided drilling tests. In all tested cases, drilling was completed successfully. An example of the picture and X-ray images are shown in Figures 12(a) and 12(b), respectively. Furthermore, the duration from algorithmic positioning calculation based on the obtained image to the commencement of drilling was approximately 2 minutes. The average execution duration for the guided drilling test was 48 seconds, and it was confirmed that the algorithm provided the necessary precision and efficiency for clinical application.

Based on the experimental results, the proposed algorithm could be applied to either approximate elliptical contours or circular contours with clinical acceptable accuracy. In addition, if the limitation of hole contour area size and the angle between two minor axes described above was fulfilled, the image contour position on the C-arm image would not affect the positioning performance. Therefore, surgeons could take and acquire C-arm images of locking holes with usual clinical procedure and then perform drilling path positioning.

## 4. Conclusion

In this paper, a drilling path-positioning algorithm was presented which could be used in distal locking of solid/slotted intramedullary nails which have at least two parallel distal locking holes. And the developed algorithm was tested in simulated, physical, and actual procedures. The data presenting the position and direction deviation and the success of the test cases proved the clinical practicality of the algorithm. In addition, in comparison with other algorithms currently used for distal locking of intramedullary nails, the entire procedure could be completed in a short time period of less than two minutes. The most significant fact was that this procedure only required one C-arm image to determine the center positions and axial directions of the two locking holes with no additional auxiliary devices, negating the requirement for repetitive imaging present in the conventional method and improving the surgical quality and efficiency. Furthermore, the guided drilling tests were done by freehand; actually, the positioning algorithm could be applied to robotic navigation system in the future for automatic drilling to assist surgeons to perform faster and more precise distal locking of intramedullary nails.
